# In Vitro and In Vivo Inhibitory Activities of Selected Traditional Medicinal Plants against Toxin-Induced Cyto- and Entero- Toxicities in Cholera

**DOI:** 10.3390/toxins14100649

**Published:** 2022-09-20

**Authors:** Rajitha Charla, Priyanka P. Patil, Arati A. Bhatkande, Nisha R. Khode, Venkanna Balaganur, Harsha V. Hegde, Darasaguppe R. Harish, Subarna Roy

**Affiliations:** 1 Indian Council of Medical Research—National Institute of Traditional Medicine, Belagavi 590010, India; 2 KLE College of Pharmacy Belagavi, KLE Academy of Higher Education and Research (KAHER), Belagavi 590010, India; 3 Indian Council of Agricultural Research—Krishi Vigyan Kendra, University of Agricultural Sciences, Bagalkot 587101, India

**Keywords:** cholera toxin, cytotoxicity, GM1 ELISA, morphological alterations, non-membrane damaging cytotoxin, cell free culture filtrate

## Abstract

*Careya arborea*, *Punica granatum*, *Psidium guajava*, *Holarrhena antidysenterica*, *Aegle marmelos*, and *Piper longum* are commonly used traditional medicines against diarrhoeal diseases in India. This study investigated the inhibitory activity of these plants against cytotoxicity and enterotoxicity induced by toxins secreted by *Vibrio cholerae.* Cholera toxin (CT) and non-membrane damaging cytotoxin (NMDCY) in cell free culture filtrate (CFCF) of *V. cholerae* were quantified using GM1 ELISA and cell-based assays, respectively. Hydro-alcoholic extracts of these plants and lyophilized juice of *P. granatum* were tested against CT-induced elevation of cAMP levels in CHO cell line, binding of CT to ganglioside GM1 receptor and NMDCY-induced cytotoxicity. Significant reduction of cAMP levels in CFCF treated CHO cell line was observed for all extracts except *P. longum. C. arborea, P. granatum, H. antidysenterica* and *A. marmelos* showed >50% binding inhibition of CT to GM1 receptor. *C. arborea, P. granatum,* and *P. guajava* effectively decreased cytotoxicity and morphological alterations caused by NMDCY in CHO cell line. Further, the efficacy of these three plants against CFCF-induced enterotoxicity was seen in adult mice ligated-ileal loop model as evidenced by decrease in volume of fluid accumulation, cAMP levels in ligated-ileal tissues, and histopathological changes in intestinal mucosa. Therefore, these plants can be further validated for their clinical use against cholera.

## 1. Introduction

*V. cholerae*, the causative agent of cholera is a facultative anaerobic gram negative, motile, non-spore forming curved rod-shaped bacteria that contains both toxigenic and non-toxigenic strains that differ in their virulence gene contents and polysaccharide surface antigens [1]. The production of Cholera toxin (CT), an 84Kd oligomeric protein efficiently secreted into the culture supernatant by the bacteria’s type 2 secretion system, is a defining feature of epidemic *V. cholerae* strains. CT is a traditional A-B toxin with a larger A subunit (CTA, catalytic domain) and five B subunits (CTB, binding domain). Its intoxication is caused by a series of events that begin with CTB binding to the GM1 receptor on intestinal epithelial cells and end with constitutive activation of adenylate cyclase, which continuously stimulates cAMP production [2]. This causes an electrolytic imbalance in enterocytes due to rapid efflux of chloride ions via the cystic fibrosis transmembrane conductance regulator (CFTR) and decreased influx of sodium ions, resulting in extensive water loss from infected cells and severe diarrhoea [3].

In addition to CT, the bacterium may secrete other potentially virulent factors, such as Accessory Cholera toxin (ACE), Zonula occludens toxin (ZOT), Heat stable enterotoxin (ST), Hemagglutinin protease (HAP), *V. cholerae* cytolysin (VCC), Haemolysin, and Non-membrane damaging cytotoxin (NMDCY) which are cytotoxic to mammalian cells to varying degrees [4,5]. Among these numerous toxins, NMDCY has a dramatic cytotoxic effect in CHO and HeLa cell lines, causing cell rounding and eventually cell death without causing membrane damage [5]. It has also been shown to have enterotoxigenic potential due to the accumulation of non-haemolytic fluid in rabbit ileal loops. It is also the most common cause of diarrhoea in non-toxigenic clinical strains of non-O1 and non-O139 *V. cholerae* [6], as well as residual diarrhoea in OCV (oral cholera vaccines) recipients [7]. According to some reports, both O1, O139 and non-O1, O139 serogroups of *V. cholerae* secrete NMDCY. The percentage positivity of NMDCY in clinical O1 strains was determined to be 81.3 percent [6], highlighting the importance of this secretogenic protein in cholera pathogenesis and piqued our interest in studying the inhibitory activity of selected plants against this cytotoxin also. Despite vast scientific evidence about other virulence factors other than CT involved in the pathogenesis of cholera, there are few reports on them. So far, studies for treating cholera have focused on either targeting bacteria or CT, but there is little scientific evidence supporting the use of cell free culture filtrate (CFCF) of *V. cholerae* containing other potential virulent factors besides CT.

Six anti-diarrhoeal plants were chosen based on their traditional use and scientific literature: *C. arborea, P. granatum, P. guajava, H. antidysenterica, P. longum* and *A. marmelos*. Unripe fruits of *A. marmelos* L. Correa., Rutaceae (Indian Bael) and leaves of *P. guajava* L., Myrtaceae (common guava) are used in traditional medicine to treat chronic diarrhoea and dysentery. Decoction of these unripe fruits and leaves has been shown to significantly reduce *Shigella flexeneri*, enteropathogenic and enteroinvasive *Escherichia coli* (EPEC, EIEC) adherence to Hep-2 cells. It also showed cidal activity against giardia, rotavirus and inhibited the binding of CT and *E. coli* labile toxin to GM1 receptor [8,9].

The seeds and bark of *H. antidysenterica* L. Wall, Apocyanaceae (Kurchi), has been shown to be effective against amoebic dysentery and *E. coli* induced diarrhoea in rats [10]. Kutajarishta is an anti-diarrhoeal formulation that contains *H. antidysenterica* as a key component. Aqueous and alcoholic bark extract showed antibacterial activity against enteric pathogens *S. flexeneri*, EIEC, EPEC and *V. cholerae***.** Fruit peel of *P. granatum* Linn., Punicaceae (Pomegranate) has traditionally been used to treat gastrointestinal disorders. It has been reported that both the peel and juice extract of pomegranate seeds have gastroprotective activity and can prevent castor oil-induced enteropooling in rats [11,12]. Bark of *C. arborea* Roxb., Lecythidaceae (wild guava) reduced castor oil-induced diarrhoea in mice significantly [13]. Piperine, a major alkaloid found in the fruits of *P. longum* (Indian long pepper), has been shown to have anti-diarrhoeal and antispasmodic properties in studies [14].

The anti-diarrhoeal activity of all six plants chosen for the study has been reported, but their specific activity against *V. cholerae*-induced diarrhoea has not been fully demonstrated. As a result, the current study investigated the inhibitory activity of these six plants on *V. cholerae* CFCF-induced cytotoxicity and enterotoxicity using in vitro and in vivo methods.

## 2. Results

### 2.1. Estimation of Polyphenolic Content

**Figure 1:**
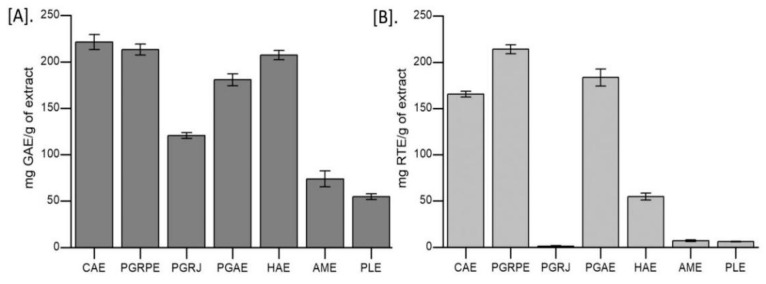
Total phenolic content (TPC) and total flavonoid content (TFC) in seven extracts (**A**). TPC in seven extracts expressed as mg gallic acid equivalents/g of extract; (**B**). TFC in seven extracts expressed as mg rutin equivalents/g of extract. CAE, *C. arborea*; PGRPE, *P. granatum* (peel extract); PGRJ, *P. granatum* (lyophilised juice); PGAE, *P. guajava*; HAE, *H. antidysenterica*; AME, *A. marmelos*; PLE, *P. longum*.

Extraction yield of seven extracts was calculated and are listed in Table S1. Based on the standard curves of gallic acid (*y* = 0.0001*x* + 0.236; *R*^2^ = 0.995) and rutin (0.0002*x* + 0.126; *R*^2^ = 0.992), TPC and TFC were estimated for 1mg of seven extracts in terms of gallic acid and rutin gram equivalents (GAE and RTE). CAE, PGRPE, and PGAE extracts contained more total phenols and flavonoids than the other extracts (Figure 1).

### 2.2. In Vitro Studies

#### 2.2.1. Cytotoxicity Assay

**Figure 2:**
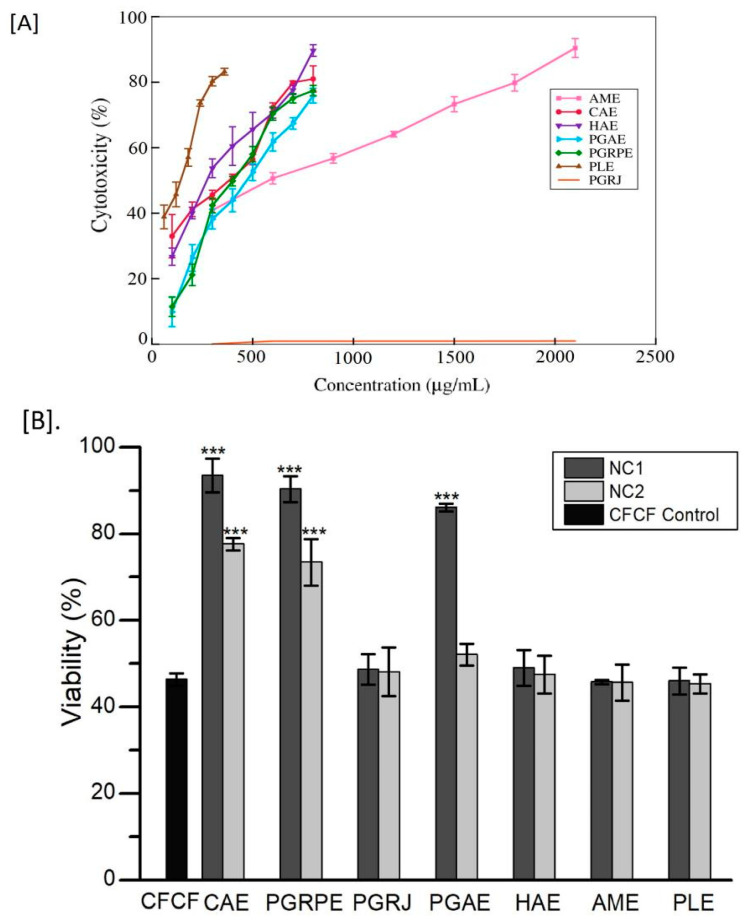
Efficacy of seven extracts against CFCF-induced cytotoxicity. (**A**) Cytotoxicity percentage of seven extracts; (**B**) Viability percentage in CFCF treated CHO cell line. CAE, *C. arborea*; PGRPE, *P. granatum* (peel extract); PGRJ, *P. granatum* (lyophilised juice); PGAE, *P. guajava*; HAE, *H. antidysenterica*; AME, *A. marmelos*; and PLE, *P. longum*. All values are expressed as a mean ± SD (*n* = 3), One Way Analysis of Variance (ANOVA) followed by Dunnett test, *** *p* < 0.001 compared with CFCF control.

Among the seven extracts, PLE had the highest toxicity with an IC_50_ of 128.9 ± 14.2 μg/mL and AME had the lowest toxicity with an IC_50_ of 646.3 ± 33.7 μg/mL, whereas PGRJ was found to be non-cytotoxic to CHO cells even at 2mg/mL lyophilised juice concentration. On CHO cells, two non-cytotoxic concentrations (NC1 and NC2) of each of the seven extracts showed a viability percentage of >90%. (Table 1). CFCF at 16-fold dilution produced 50% viability in CHO cell line using MTT assay and was considered CFCF IC_50_ titre (Figure S1). In the following experiment, CHO cells were treated with a pre-incubated mixture of NC1 and NC2 from each of the seven extracts, as well as the IC_50_ titre of CFCF. Significant increase in viability% (*** *p* < 0.001) was observed for CAE, PGRPE, and PGAE compared with CFCF control (Figure 2).

#### 2.2.2. Binding Inhibition of CT to GM1

**Figure 3:**
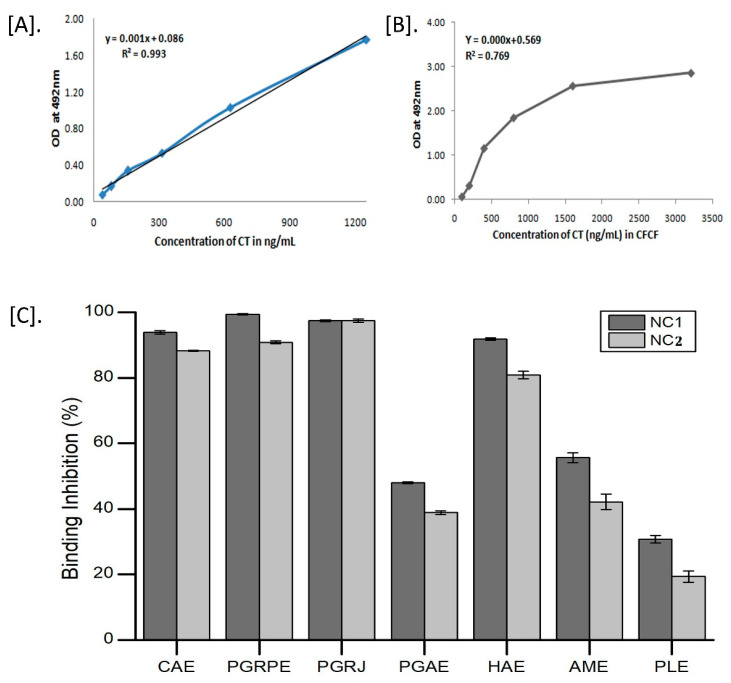
Determination of CT concentration in CFCF and its binding inhibition to GM1. (**A**) Standard graph of CT; (**B**) Absorbance curve of CT in CFCF; (**C**) Binding inhibition percentage (BI%) of CT to GM1 in presence of seven extracts using GM1 ELISA.

Each batch of CFCF was prepared fresh, and CT concentration was estimated using ganglioside GM1 ELISA, yielding an average CT concentration of 18.76 µg/mL ± 0.2 using the CT standard curve. The highest absorbance of CFCF titre with 1600 ng/mL of CT (1:5 dilution) indicated GM1 saturation with CT. CAE, PGRPE, PGRJ, and HAE showed >90% BI percent of CT to GM1 in a pre-incubated mixture of plant extracts (NC1 and NC2) with CFCF titre (Figure 3).

#### 2.2.3. Cyclic AMP Assay

**Figure 4:**
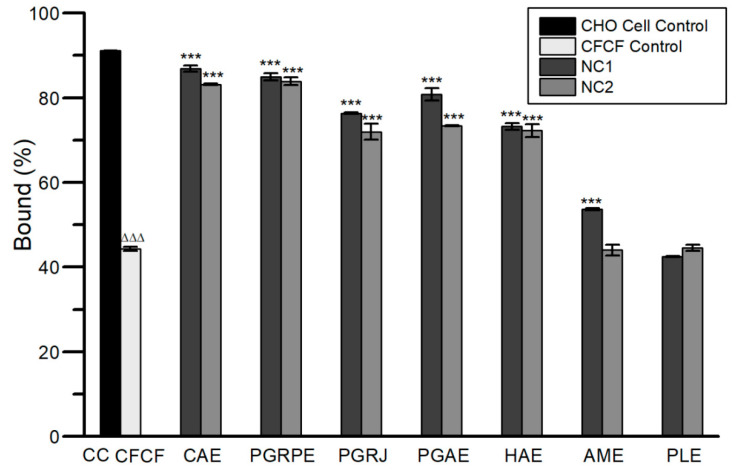
Effect of seven extracts on increased cAMP levels in CFCF-induced CHO cell line. All values were expressed as mean ± SD, One Way Analysis of Variance (ANOVA) followed by Dunnett test. ^ΔΔΔ^ *p* < 0.001, compared with CHO cell control. *** *p* < 0.001 compared with CFCF control.

Cyclic AMP levels were decreased significantly (*** *p* < 0.001) for CAE, PGRPE, PGRJ, PGAE and HAE at both NC1 and NC2 except for AME when compared to CFCF control. Among the seven extracts, PLE showed no activity against elevated cAMP levels in CHO cells (Figure 4).

#### 2.2.4. Protective Activity against Morphological Alterations

**Figure 5:**
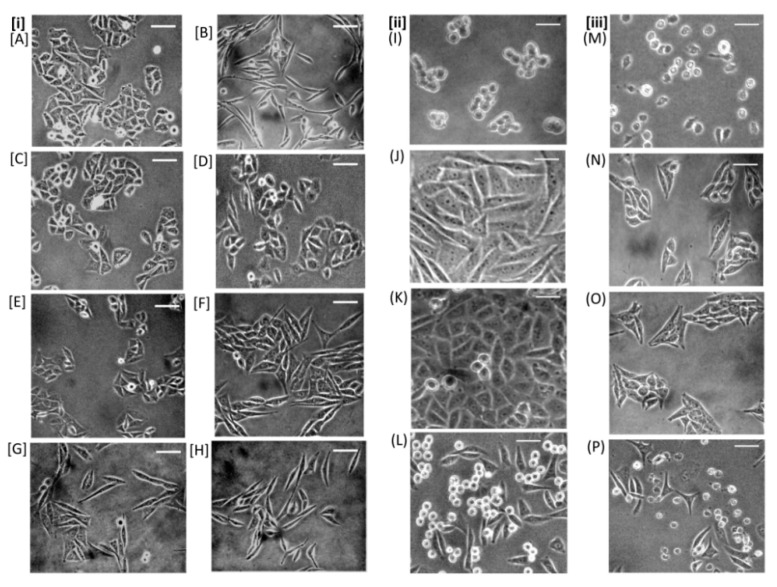
Protective activity of seven extracts (NC1) against morphological alterations. (**i**) CHO cell control (**A**), CHO cells treated with CFCF TCID_100_ for cell elongation (CFCE:64-fold dilution of CFCF) (**B**), CAE + CFCE (**C**), PGAE + CFCE (**D**), PGRPE + CFCE (**E**), PGRJ + CFCE (**F**), HAE + CFCE (**G**), AME + CFCE (**H**). (**ii**) CHO cell control treated with CFCF TCID_100_ for cell rounding (CFCR:8-fold dilution of CFCF) (**I**), CAE + CFCR (**J**), PGRPE + CFCR (**K**), PGAE + CFCR (**L**). (**iii**) CHO cells control treated with CFCF TCID_100_ for cell death (CFCD: 2-fold dilution of CFCF) (**M**), CAE + CFCD (**N**), PGRPE + CFCD (**O**), PGAE + CFCD (**P**) using phase contrast microscopy at 40× magnification (scale bar-5 µm).

TCID_100_ was determined as reciprocal of highest dilution of CFCF showing three levels of morphological changes in CHO cell line viz. cell elongation (CE), cell rounding (CR), and cell death (CD). TCID_100_ for CE, CR, and CD were estimated as 64-fold, 8-fold, and 2-fold dilutions of CFCF, respectively. Except for PLE, all of the selected extracts inhibited cell elongation induced by CT in the CHO cell line (Figure 5i(A–H)), whereas three plant extracts, namely CAE, PGRPE, and PGAE demonstrated effective protection against NMDCY-induced cell rounding (Figure 5ii(I–L)), as well as cell death (Figure 5iii(M–P)) in comparison to the CFCF control.

### 2.3. In Vivo Studies

#### 2.3.1. Mice Ligated-Ileal Loop Assay

**Figure 6:**
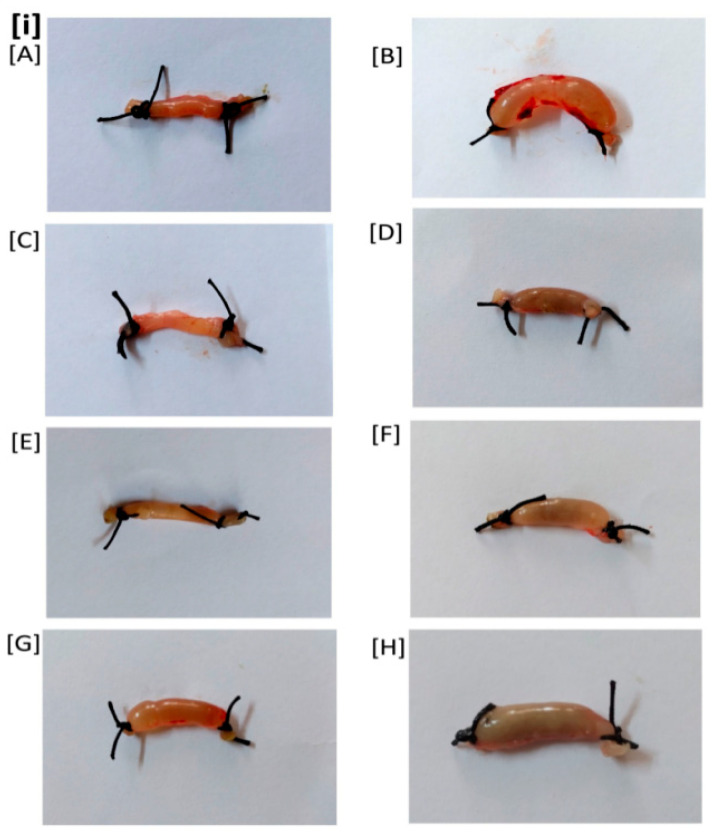

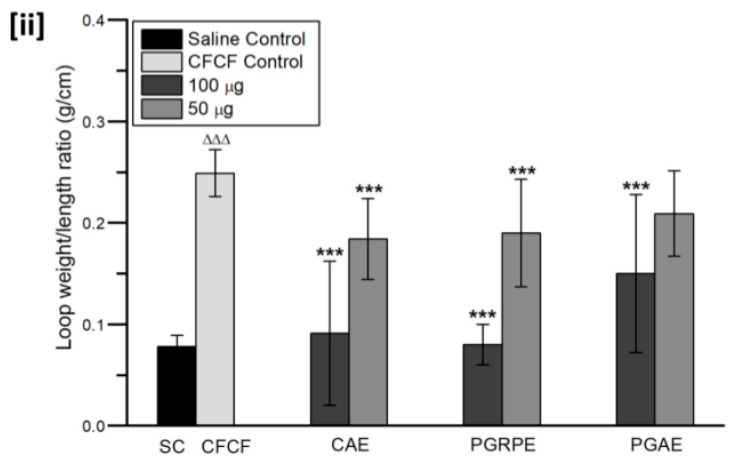
Protective activity of plant extracts against CFCF-induced fluid accumulation using mice ligated-ileal loop assay. (**i**) (**A**) Saline control (100 μL PBS), (**B**) CFCF control (CT = 1 μg/loop), (**C**) CAE (100 μg) + CFCF, (**D**) CAE (50 μg) + CFCF, (**E**) PGRPE (100 μg) + CFCF, (**F**) PGRPE (50 μg) + CFCF, (**G**) PGAE (100 μg) + CFCF, (**H**) PGAE (50 μg) + CFCF. (**ii**) Weight/length ratio of mice ligated-ileal loops; All values were expressed as mean ± SEM (*n* = 6) One Way Analysis of Variance (ANOVA) followed by Dunnett test. ^ΔΔΔ^ *p* < 0.001, compared with saline control. *** *p* < 0.001 compared with CFCF control.

Based on the in vitro findings, two concentrations (100 µg and 50 µg/loop) of three extracts CAE, PGRPE, and PGAE against CFCF containing 1 µg of CT were chosen for further in vivo testing. The mean weight/length ratio of CFCF treated ileal loops was 0.249 g/cm ± 0.02 while that of untreated saline instilled ileal loops was 0.078 g/cm ± 0.01. It was discovered that mice ileal loops treated with CFCF containing 1 µg of CT caused significant intestinal fluid accumulation after 18 h of incubation (^ΔΔΔ^ *p* < 0.001) when compared to the Saline control group. CAE and PGRPE at both concentrations resulted in a significant reduction in intestinal fluid accumulation in all test groups (*** *p* < 0.001) when compared to the CFCF control group (Figure 6). Table S2 shows the mean W/L ratios of the control and test groups.

#### 2.3.2. Estimation of cAMP Levels in Ligated-Ileal Loops

**Figure 7:**
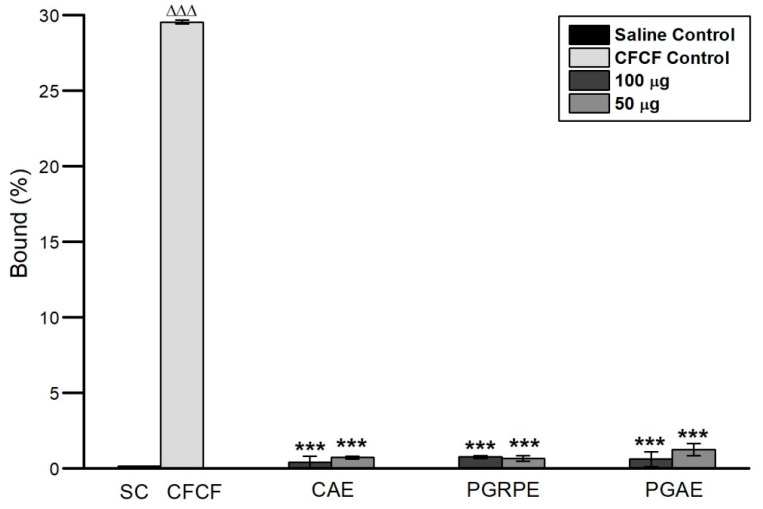
Cellular cAMP levels in ligated-ileal tissues. All values were expressed as mean ± SD, One Way Analysis of Variance (ANOVA) followed by Dunnett test. ^ΔΔΔ^ *p* < 0.001, compared with Saline control. *** *p* < 0.001 compared with CFCF control.

When compared to the Saline control group, there was a significant increase in cAMP levels in the CFCF control (^ΔΔΔ^ *p* < 0.001). CAE, PGRPE, and PGAE inhibited CFCF-induced cAMP levels in ileal tissues significantly (*** *p* < 0.001), as evidenced by their reduction in cellular cAMP levels (Figure 7).

#### 2.3.3. Histopathology

**Figure 8:**
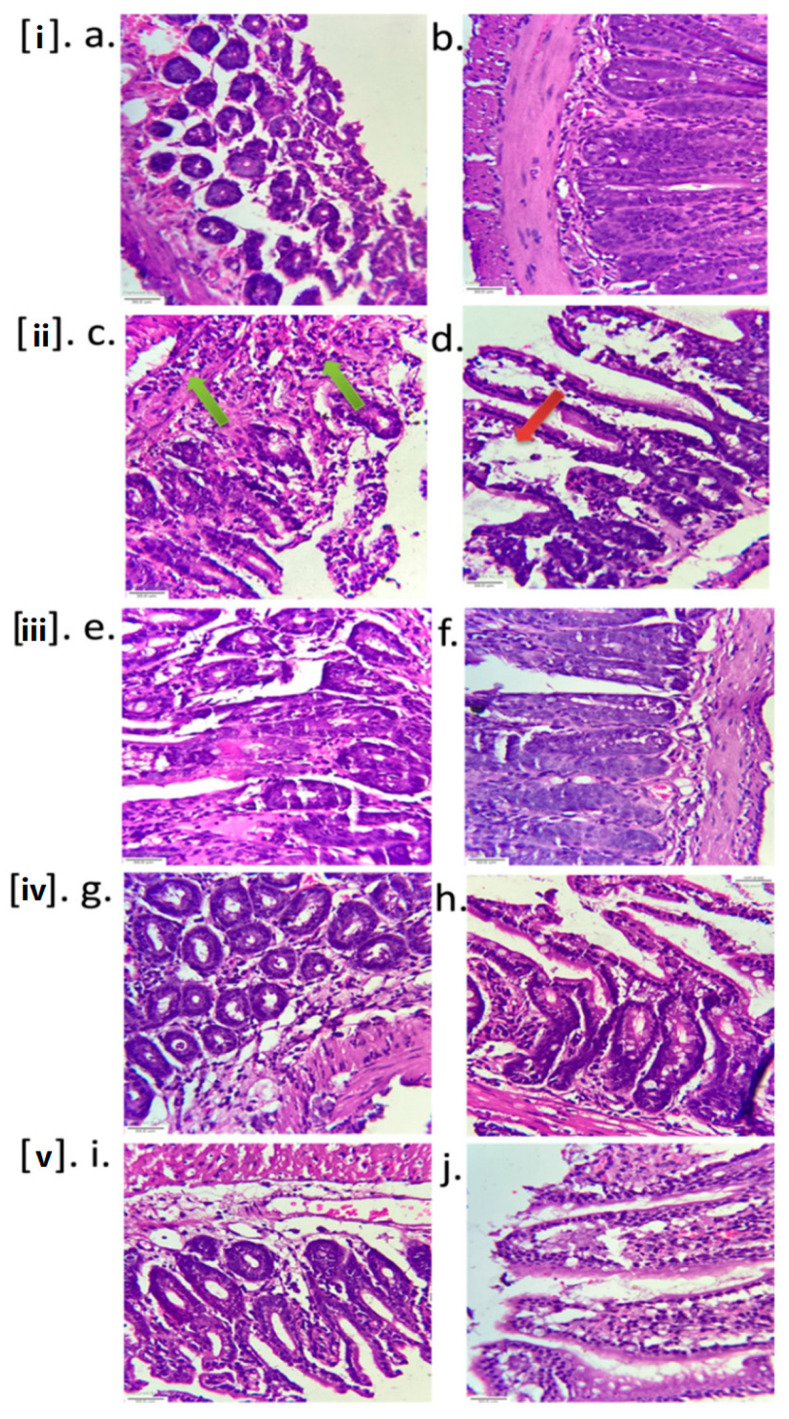
Histopathological analysis of mice ileal loops. Intestinal tissues of mice ileal sections were stained with hematoxylin and eosin and observed at 40× magnification (scale bar = 30 µm). Inflammation in submucosa represented in green arrows (**ii**(**c**)) and mucosal layer degeneration, diffused edema represented in red arrows (**ii**(**d**)). (**i**(**a**,**b**)) Saline control; (**ii**(**c**,**d**)) CFCF control (CT = 1 µg); (**iii**(**e**,**f**)) CAE (100 µg) + CFCF (CT = 1 µg); (**iv**(**g**,**h**)) PGRPE(100 µg) + CFCF(CT = 1 µg); (**v**(**i**,**j**)) PGAE(100 µg) + CFCF (CT = 1 µg).

Histological examination of CFCF-treated ileal loops revealed significant changes in intestinal tissues when compared to the Saline control group. After 18 h of CFCF administration into ligated-ileal loops, there was prominent inflammatory cellular infiltration in the submucosa (green arrows, Figure 8ii(c)), as well as further mucosal layer degeneration and diffused edema in the intestinal villi (red arrows, Figure 8ii(d)). The morphology of the mucosal and submucosal layers was normal in the Saline control group. Among the treated groups, 100 µg of CAE and PGRPE greatly reduced the histopathological changes induced by CFCF in mice ileal loops as indicated by the decreased infiltration inflammatory cells and edema. In other treated groups, amelioration of histological changes was observed to lesser extent compared to 100 µg of CAE and PGRPE.

## 3. Discussion

This study utilised the cell free culture filtrate of O1 El Tor Ogawa *V. cholerae* isolated from clinical samples during the cholera out-breaks in Karnataka, India [15,16] for both in vitro as well as in vivo studies. It has been well reported that O1 El Tor Ogawa strain of *V. cholerae* produce Cholera toxin [17], non-membrane damaging cytotoxin [18] into culture supernatant along with other toxins such as Accessory Cholera toxin [19], Zonula occludens toxin [20], Multifunction auto processing repeats-in-toxin [21], Haemolysin [22] which are responsible for the pathogenesis of *V. cholerae*. Previous research has shown that the bacterium efficiently secretes both CT and NMDCY in AK1 media [23,24]. When grown in AK1 media, the clinical strain of *V. cholerae* used in this study produced both CT and NMDCY in the culture filtrate. Using GM1 ELISA, the concentration of CT in CFCF was estimated to be 18.76 µg/mL ± 0.2, whereas NMDCY demonstrated IC50 on CHO cell line at 16-fold dilution of CFCF using MTT assay. CT and NMDCY both have cytotoxic effects on the CHO cell line; CT increases cAMP levels and changes cell morphology in the CHO cell line. Cell elongation observed in CFCF-treated CHO cells is a distinct effect of the bacterium’s CT secreted into CFCF. This elongation morphology observed in CT-treated CHO cells is due to elevated cAMP levels caused by permanent ADP-ribosylation of Gsα and activation of adenylate cyclase, which continuously stimulates cAMP production [25]. In the CHO cell line, 100 ng/mL of CT in CFCF caused cell elongation and a significant increase in cellular cAMP levels; an earlier study by Saha et al., [26] found similar results.

NMDCY secreted into CFCF produced cell rounding and cell death in CHO cell line as reported in a similar study by Saha et al., [18]. The morphological changes observed in CFCF-treated CHO cells in our study were strikingly similar to those observed in NMDCY-induced cellular intoxication in CHO and HeLa cells. Evidently, with further increase in concentration of CFCF, CHO cells switched from cell rounding to cell death without accompanying the membrane damage as observed under the phase contrast microscope (Figure 5ii(I),iii(M)). Cell death is caused by a series of events that include major changes in cellular synthetic, secretory, and cell cycle dependent functions during the earlier phase of NMDCY intoxication and cytoskeleton disorganization during the later phase [5]. As previously reported, the assay for cytotoxic activity of NMDCY on CHO cell line producing cell rounding and cell death was performed using 100% tissue culture infective dose (TCID_100_) and direct observation under phase contrast microscope [6]. The reciprocal of the highest dilution of CFCF producing a 100% cytotoxic effect on the CHO cell line was used to calculate TCID_100_. The current study demonstrated 100% cell rounding and cell death in CHO cell lines at 8-fold and 2-fold dilutions of CFCF, respectively, whereas an earlier study by Saha et al., [6] demonstrated 100% cell rounding at 16-fold dilutions of CFCF.

Seven extracts from six plants (*A. marmelos*, *P. guajava*, *H. antidysenterica*, *C. arborea*, *P. granatum*, and *P. longum*) were chosen for this study based on their traditional use and anti-diarrhoeal activity. Their efficacy against CFCF-induced cellular toxicities was investigated using non-cytotoxic concentrations (NC1 and NC2) that demonstrated >90% cell viability on the CHO cell line. The first and most important step in CT intoxication is the binding of its B subunit to the GM1 receptor on intestinal epithelial cells, which allows CT to be transported retrogradely into the cytosol [27]. A subunit of CT that enters the enterocyte’s cytosol has ADP-ribosyl transferase activity and catalyses the permanent ADP-ribosylation of Gsα. This results in continuous stimulation of adenylate cyclase and rise in cellular cAMP levels. Elevated cAMP levels in the host cell activates cystic fibrosis transmembrane conductance regulator (CFTR) causing dramatic efflux of ions and water from infected enterocytes leading to diarrhoea [28]. As CT binds with ganglioside GM1 with high affinity and specificity, we investigated the inhibition of CT binding to GM1 (BI%) by selected seven extracts using GM1 ELISA. Among these six plants, *A. marmelos* fruits and *P. guajava* leaves showed 55% and 40% binding percentage of CT to GM1 with 10% of their respective decoctions in an earlier study [8,9]. In our study, hydro-alcoholic extracts of *A. marmelos* and *P. guajava* showed 55% and 48% binding inhibition of CT to GM1 at NC1, respectively. However, no previous reports for other plants were discovered. At NC1 and NC2, the BI% of the other four plants was estimated to be CAE (94%, 88%), PGRPE (99%, 91%), PGRJ (97%, 97%), HAE (92%, 81%), and PLE (31%, 19%). The GM1 ELISA results show that, with the exception of PLE, all other extracts exhibited anti-CT activity by inhibiting CT binding to the GM1 receptor.

Using competitive cAMP ELISA, the efficacy of seven extracts against CT-induced elevation of cAMP in CHO cell line was evaluated as increase in bound percent (B%) of cAMP (Figure 4). From selected seven, five extracts showed significant increase (*p* < 0.001) in B% i.e., CAE (87%, 83%), PGRPE (85%, 84%), PGRJ (76%, 72%), PGAE (81%, 73%) and HAE (73%, 72%) at NC1 and NC2, respectively compared with CFCF control (44%). The cyclic AMP assay results were related to the protective activity of seven extracts against CT-induced cell elongation in CHO cells. The protection against CT-induced cell elongation in CHO cells was observed to be prominent in the presence of CAE, PGRPE, PGRJ, PGAE, HAE, and AME, which was consistent with the results from the cAMP assay (Figure 5i(A–H)). The cumulative results of in vitro studies corresponding to anti-CT activity of seven extracts conclude that, with the exception of PLE, all other extracts showed varying degrees of neutralizing activity against CT validating the traditional use of these plants in treating CT-induced diarrhoeal infections.

The MTT assay was used to determine the counteracting activity of seven extracts against NMDCY-induced cytotoxicity. CFCF control (16-fold dilution) showed 50% cell viability in CHO cell line and two plant extracts CAE (93%, 78%), PGRPE (90%, 73%) showed significant defensive activity (*p* < 0.001) against NMDCY induced cytotoxicity at NC1 and NC2 (Figure 2) whereas PGAE (86%, 52%) showed significant protective activity at NC1. The MTT assay results were found to be correlated with the protective activity of seven extracts against NMDCY-induced morphological changes in the CHO cell line. CAE, PGRPE, and PGAE also demonstrated effective protection against NMDCY-induced cell rounding and cell death in the CHO cell line, whereas PGRJ, HAE, AME, and PLE had no activity against NMDCY.

According to our in vitro studies with *V. cholerae* CFCF, there is differential activity of seven extracts against CT and NMDCY induced cellular toxicities. Except for PLE, all other plants had anti-CT activity, whereas only CAE, PGRPE, and PGAE had NMDCY neutralizing activity. Interestingly, there was a correlation between total phenolic and total flavonoid content in extracts and CT binding inhibition to GM1, decreased CT-induced elevated cAMP levels, and NMDCY induced cell rounding in CHO cells. The plants with the highest phenolic content, i.e., CAE, PGRPE, PGRJ, and HA had more than 90% BI percent of CT to GM1, whereas plants with higher flavonoid content, i.e., CAE, PGRPE, and PGAE were found to be more resistant to CT-induced cAMP and NMDCY-induced cytotoxicity. The current study supports the efficacy of hydro alcoholic extracts of *C. arborea*, *P. granatum*, and *P. guajava* against cytotoxicity induced by CT as well as NMDCY secreted by *V. cholerae*. As a result, the efficacy of these extracts against enterotoxicity was investigated further using an adult mouse ligated-ileal loop model

CT has strong enterotoxigenic activity and causes fluid accumulation in ligated -ileal loops of adult mice [29]. CFCF containing 1 μg of CT when instilled into distal ligated-ileal loops of 2–3 cm in length, elicited fluid accumulation after 18hrs and loop mean (*n* = 6) weight/length ratio was estimated 0.25 g/cm ± 0.023 whereas a similar study by Sawasvirojwong et al., [30] showed 0.192 ± 0.042 g/cm. This fluid accumulation induced by CFCF was observed to be significant (*p* < 0.001) compared with saline control group (0.09 ± 0.1 g/cm). A pre-incubated CFCF mixture containing 1 μg of CT and three extracts at two different concentrations (100 μg, 50 μg) reduced CFCF-induced fluid accumulation significantly (*p* < 0.001) (Figure 6). Using competitive ELISA, the cellular cAMP levels in each group’s pooled ileal tissues were estimated as an increase in bound percent (B%). Significant increase in cAMP levels (*p* < 0.001) were observed in CFCF control group (29.5%) compared to Saline control (0.1%). CAE (0.41%, 0.73%), PGRPE (0.76%, 0.66%) and PGAE (0.6%, 1.2%) showed significant reduction in cAMP levels (*p* < 0.001) at 100 µg and 50 µg respectively (Figure 7). Histopathological analysis of CFCF-treated ileal loops revealed inflammation in the submucosa and diffused edema in the mucosal layer of the intestine, and similar findings have previously been reported [30]. Inflammation and edema were significantly reduced in CFCF-treated ileal loops in the presence of CAE and PGRPE. However, when compared to the CFCF control, PGAE showed less improvement in histopathological changes (Figure 8). Previous research with tea polyphenols [31] and apple polyphenols [29] reported to decrease CT-induced fluid accumulation in rabbits and adult mice ligated-ileal loop reflected the anti-diarrhoeal effect of polyphenols in these plant extracts. The higher polyphenolic content of CAE and PGRPE could explain their effective enterotoxic activity against CT-induced fluid accumulation.

## 4. Conclusions

The current study investigated the efficacy of six anti-diarrhoeal plant extracts against *V. cholerae* CFCF-induced toxicity, including their inhibitory activity against CT and NMDCY. Among the selected six plants, the hydro alcoholic extracts of *C. arborea*, *P. granatum*, and *P. guajava* were found to be effective against CT and NMDCY. In vivo studies with these plants reduced CFCF-induced fluid accumulation, cellular cAMP levels, and histopathological changes in ligated-ileal loops of adult mice. Our study demonstrates the use of these three plant extracts as a supplement to regular oral rehydration therapy in the treatment of cholera, and it is also expected to reduce the excessive use of antibiotics against these enteropathogens in the treatment of the infection in a cost-effective manner with health and environmental benefits. More clinical research is needed to validate the use of these plants against *V. cholerae*-induced diarrhea.

## 5. Materials and Methods

### 5.1. Materials

Cholera toxin, anti-Cholera toxin antibody, anti-rabbit IgG-peroxidase, monosialoganglioside GM1, o-Phenylenediamine were procured from Sigma Aldrich (St. Louis, MI, USA). CHO (Chinese Hamster Ovary) cell line was obtained from NCCS, Pune. Dulbecco’s Modified Eagle Medium (DMEM), Fetal Bovine Serum, pencillin-streptomycin, and amphotericin B were purchased from Thermo Fischer Scientific (Waltham, MA, USA).

### 5.2. Plant Collection, Authentication and Preparation of Extract

Unripe fully matured fruits of *A. marmelos*, bark of *C. arborea* and *H. antidysenterica*; young leaves of *P. guajava*; fruits of *P. granatum* and *P. longum* were collected in the Belagavi district of Karnataka, India. A plant taxonomist at ICMR-NITM, Belagavi, identified and certified the collected plants and their parts. The voucher specimens were deposited in ICMR-NITM under accession numbers 1590 (*C. arborea*), 1690 (*P. granatum*), 1589 (*P. guajava*), 1591 (*H. antidysenterica*), 1588 (*A. marmelos*), and 1691 (*P. longum*).

*P. longum* fruits were defatted using 10× petroleum ether by Soxhlet apparatus prior to extraction. All the six dried and powdered plant materials (100 g) *viz.* deffated fruits of *P. longum*, bark of *C. arborea* and *H. antidysenterica*, leaves of *P. guajava,* fruits of *A. marmelos* and fruit peel of *P. granatum* except seeds of *P. granatum* were mixed with five times the amount of 70% ethanol (*v*/*v*) in water and extracted using the cold maceration technique [32]. The juice prepared from *P. granatum* seeds was filtered and lyophilised. After calculating the extraction yield, the seven extracts from six plants were stored at −20 °C for future use. 

### 5.3. Estimation of Polyphenolic Content

As polyphenols were reported to show anti-CT activity [33], total phenolic content and total flavonoid content in seven extracts were estimated using Folin-Ciocalteu [34] and AlCl_3_ colorimetric methods [35], respectively, with minor modifications. 

#### 5.3.1. Phenolic Content in Total (TPC)

A 1:20 dilution of the Folin-Ciocalteu reagent in distilled water was added to seven extracts taken at three different concentrations (500 µg, 1 mg, and 2 mg/mL). To this, 4 mL of 7.5% Na_2_CO_3_ and distilled water were added to make the total volume 15 mL. This mixture was allowed to stand at room temperature for 2 h before measuring absorbance at 750 nm. TPC was measured in milligrams of gallic acid equivalents per gram of dry extract. 

#### 5.3.2. Flavonoid Total Content (TFC)

Seven extracts of varying concentrations (500 µg, 1 mg, and 2 mg/mL) were added to a volumetric flask containing 4mL distilled water, and this mixture was treated with 0.3 mL NaNO_2_. After 5 m, 0.3 mL of 10% AlCl_3_ was added, followed by 0.2 mL of 1M NaOH at the 6th minute. Dis.H_2_O was used to make a total volume of 10 mL, and absorbance was measured at 510 nm. TFC was measured in milligrams of rutin equivalents per gram of dry extract. 

### 5.4. In Vitro Studies

#### 5.4.1. CFCF Preparation

The clinical isolates of *V. cholerae* were collected from different regions of the southern Indian state of Karnataka with cholera outbreaks in 2010 and 2012 [15,16]. All of these isolates are serotype O1 El Tor Ogawa and have been preserved as glycerol stocks at the ICMR-NITM microbial strain repository. A single isolate of *V. cholerae* was chosen based on CT production determined by GM1 ELISA and cell rounding determined in the CHO cell line at the highest dilution. 

Because both CT and NMDCY have been shown to secrete efficiently in AK1 media, *V. cholerae* were cultured in AK1 medium to induce virulence gene expression, as well as the production of NMDCY and CT, as previously described [23,24]. The bacteria were first streaked on Mueller-Hinton agar medium, and a single colony was picked and inoculated in MH broth for 12 h. The inoculum size was adjusted to 10^5^–10^7^ cfu/mL, and 1mL of inoculum was added to 10mL of AK1 medium in stationary tubes and incubated at 37 °C for 4 h. The culture was then transferred to a flask and shaken at 150 rpm for 16 h. To pellet the bacterial cells, the culture fluid was centrifuged at 12,000 rpm for 20 m at 4 °C. The cell free supernatant was filtered through a 0.22 μm syringe filter and stored at −20 °C as cell free culture filtrate (CFCF) for both in vitro and in vivo experiments. 

#### 5.4.2. CT Concentration Estimation in CFCF

The amount of CT secreted into CFCF was calculated using the GM1 ELISA as previously described [36,37]. The standard curve was plotted using pure CT reconstituted at 5 mg/mL in deionised water. In brief, CT taken at known concentration from 1250 ng/mL was diluted in two-folds up to 39 ng/mL and 100 μL of CFCF at different dilutions (1:5; 1:10; 1:20 and 1:30) in PBS (pH 7.4) were added to GM1 (1.44 M) pre-coated on 96-well microtitre plate. To provide a baseline measurement, distilled water was used as the solvent control for CT and AK1 media for CFCF. The total volume in each well was made to 100 μL with PBS and incubated at 37 °C for 1 h. The wells were then washed three times in PBS buffer with 0.05 percent Tween20. ELISA was performed using polyclonal anti-CT antibody produced in rabbit diluted 1:600 in PBS and anti-rabbit IgG-peroxidase diluted 1:200 in PBS. To visualise CT binding to GM1, 50 μL of citrate buffer (pH-5.5) containing 0.04 percent (*w/v*) OPD and 0.2 percent (*v*/*v*) hydrogen peroxide was added to each well and incubated for 5 m in the dark at room temperature. To stop the reaction, 50 μL of 2.5N H_2_SO_4_ was added, and absorbance at 492 nm was measured using a UV/Vis microplate spectrophotometer (Multiskan GO, Thermo Scientific). The CT standard curve was used to estimate the concentration of CT secreted in CFCF.

#### 5.4.3. Cytotoxicity Assay

MTT assay was used to determine the cytotoxicity of seven extracts and CFCF on CHO cell line [38]. At 37 °C and 5% CO_2_, CHO cells were cultured in DMEM medium supplemented with 10% FBS, pencillin-streptomycin, and amphotericin b. Stock solutions of seven extracts were prepared at 10mg/mL in 5% DMSO and filtered through a 0.22 μm syringe filter. CFCF prepared as previously described was serially diluted in DMEM media containing 2% FBS. CHO cells were seeded at a density of 2 × 10^4^ per well in a 96-well plate and incubated for 24 h at 37 °C and 5% CO_2_. After 24 h, cells were treated in triplicate with increasing concentrations of seven extracts ranging from 60 μg to 2 mg/mL and twofold dilutions of CFCF. Untreated cells and cells treated with DMSO/AK1 media were taken as cell control and solvent control respectively. The total volume in each well was made to 200 μL with DMEM supplemented with 2% FBS and incubated for 24 h The cells were then washed with PBS and treated with 20 μL of 0.5 percent MTT solution in PBS and incubated for 4 h. After 4 h, the formazan crystals were solubilized with 100 μL of DMSO. The absorbance was measured at 570 nm and the cytotoxicity percentage was calculated. 

In the following experiment, the two non-cytotoxic concentrations (NC1 and NC2) of all seven plant extracts were chosen by using 25% and 12.5% of their respective IC_50_ concentrations that demonstrated >90% cell viability using the MTT assay. Furthermore, NC1 and NC2 from all seven extracts were pre-incubated with CFCF IC_50_ titre for 4 h at 120 rpm at 37 °C before being treated with CHO cells for 24 h. Untreated cells served as the cell control, and CFCF-treated CHO cells served as the CFCF control. The MTT assay was carried out as previously described, and the protective activity of seven extracts against CFCF-induced cytotoxicity was expressed as percentage of viability. 

#### 5.4.4. Binding Inhibition of CT to Ganglioside GM1

The inhibition of CT binding to GM1 by seven extracts was assessed using GM1 ELISA as previously described [36,37]. The CFCF titre with CT concentration 3200 ng/mL diluted two-fold upto 100 ng/mL was initially used to plot CFCF absorbance curve and to identify the CT concentration in CFCF with highest absorbance at highest dilution. The selected CFCF titre was treated with NC1 and NC2 of each seven plant extracts in PBS and incubated for 4 h on shaker at 120 rpm at 37 °C. The pre-incubated mixture was added to pre-coated GM1 96-well micro titre plates in triplicates. Each concentration of seven plant extracts was used as a negative control, while CFCF alone was used as a positive control. The mean absorbance of CFCF was used as a control, and the percentage of binding inhibition was calculated as follows. 

Binding inhibition percentage (BI%) = (1−A_test_ ÷ A_CFCF control_) × 100

#### 5.4.5. Cyclic AMP Assay

Cyclic AMP levels in CFCF-induced CHO cells were determined using the cAMP in vitro competitive ELISA kit (Abcam, Waltham, MA, USA). CHO cells were seeded at 4 × 10^4^ cells per well in a 96-well flat bottom microplate and incubated for 24 h at 5% CO_2_ and 37 °C. Cells were washed after 24 h and treated with a pre-incubated mixture of seven extracts at two non-cytotoxic concentrations and CFCF with a CT concentration of 100 ng/mL. The volume in each well was made to 200 µL with DMEM supplemented with 2% FBS and incubated for 18 hrs. After 18 h, the cell lysate was prepared by treating the cells with 0.1M HCl and 1% tritonX-100. Under a microscope, uniform lysis of cells was observed, and lysate supernatant was obtained by centrifuging cell lysate at 2000 rpm. Cell lysates from test samples were added in duplicate to the kit wells, and the assay was carried out according to the instructions. Untreated cells, CFCF-treated cells, and cells treated with each of the seven extracts separately served as controls. Cyclic AMP levels were calculated as a percentage of bound cAMP (B%) for cells treated with a combination of seven extracts/CFCF and control cells treated with each of the seven extracts/CFCF/DMEM. 

#### 5.4.6. Protective Activity against Morphological Changes

The morphological changes in the CFCF-induced CHO cell line were observed directly with an inverted phase-contrast microscope, as described [18]. CHO cells were seeded at 6 × 10^4^ in 12-well plate culture dishes with DMEM supplemented with 10% FBS and incubated for 24 h at 5% CO_2_ and 37 °C. The cells were washed before being treated with equal volumes of twofold serial dilutions of CFCF in DMEM with 2% FBS. After 24 h, cells were examined under a phase contrast microscope at 40× magnification for morphological changes. As a negative control, CHO cells were treated with similar dilutions of AK1 media. The reciprocal of the highest CFCF dilution at which 100% of CHO cells showed three levels of morphological changes i.e., CT-induced cell elongation, NMDCY-induced cell rounding, and cell death were quantified and taken as a 100 percent tissue culture infective dose (TCID_100_). Each of the seven extracts at two concentrations, NC1 and NC2 are treated with TCID_100_ of CFCF, resulting in three different morphological alterations and incubated on a shaker at 120 rpm for 4 h at 37 °C. This CFCF pre-incubated with the extracts was then treated with CHO cells seeded at 6 × 10^4^ in a 12-well plate in duplicate; cells treated with only CFCF served as a positive control, and untreated cells served as a cell control. The cells were then incubated for 24 h at 5% CO_2_ and 37 °C before the media was discarded and the cells were washed. The cells were replenished with fresh media, examined under a phase contrast microscope for morphological changes, and photographed at 40× magnification. 

### 5.5. In Vivo Studies

Based on in vitro results, CAE, PGRPE and PGAE were investigated further using in vivo studies.

#### 5.5.1. Mice Ligated-Ileal Loop Assay

The ligated-ileal loop assay was performed on adult Swiss albino mice to assess the protective activity of CAE, PGRPE, and PGAE against CFCF-induced fluid accumulation [30]. Animal experiments on adult mice were approved by Institutional Animal Ethical Committee of ICMR-NITM, Belagavi (Reg No: 1388/GO/Re/S/10/CPCSEA). Six-week-old Swiss albino mice weighing 25–30 g were obtained from the MMDC laboratory animal facility in Belagavi, Karnataka, and acclimatised under controlled temperature, humidity, and light conditions at the ICMR-NITM Animal Research Facility in Belagavi. 

A total of 48 Swiss albino mice were randomly assigned to one of eight groups (*n* = 6), as follows: (A) Saline control (Untreated): received 100 μL of saline/loop; (B) CFCF control: received CFCF with CT concentration (1 µg/loop); (C) CAE1: 100 µg of CAE +CFCF (CT 1 µg)/loop; (D) CAE2: 50 µg of CAE + CFCF (CT1 µg)/loop; (E) PGRPE1: 100 µg of PGRPE + CFCF (CT 1 µg)/loop; (F) PGRPE2: 50 µg of PGRPE + CFCF(CT 1 µg)/loop; (G) PGAE1: 100 µg of PGAE + CFCF(CT 1 µg)/loop; (H) PGAE2: 50 µg of PGAE + CFCF (CT 1 µg)/loop. Each ligated-ileal loop in groups C–H received a pre-incubated mixture of three plant extracts at two different concentrations with CFCF. 

The mice were fasted overnight prior to the experiment and given free access to water before being anaesthetized with an intraperitoneal injection of Ketamine-Xylazine (87.5 mg/kg; 12.5 mg/kg). A small abdominal incision was made through which a 2–3 cm long loop of distal ileum was exteriorized and tied at both ends with surgical thread. The sealed ileal loops were then injected with PBS/CFCF/plant extracts + CFCF without causing injury to the tissue, gently restituted and sutured. These mice were then allowed to recover from anaesthesia before being placed in appropriate cages with free access to food and water. Mice were sacrificed after 18 h by overdosing on anaesthesia, and ileal loops were excised from the carcass. To determine the protective activity of selected three extracts against CFCF-induced fluid accumulation in mice, the length and weight of the ileal loops were measured, and mean weight/length ratio was calculated. 

#### 5.5.2. Estimation of cAMP Levels in Ligated-Ileal Loops

Cellular cAMP levels in ileal tissue were measured using an abcam competitive ELISA kit as directed by the manufacturer. After 18 h, ligated-ileal loops were excised from sacrificed mice and stored at −80 °C. The weight of ileal tissues from each group (A to H) was determined. Homogenisation of 100 mg of pooled ileal tissues from each group was performed with lysis buffer, and supernatant was collected by centrifuging at 12,000 rpm for 10 min at 4 °C and cAMP levels were determined. 

#### 5.5.3. Histopathology

Excised ileal loops were histopathologically examined after being fixed in 10% formalin. The intestinal tissues were stained using Hematoxylin and Eosin reagents and observed at 40× magnification. The histological changes were analysed in control and treated groups as described by Sawasvirojwong (2013).

### 5.6. Statistical Analysis

The results of in vitro assays were expressed in terms of mean ± standard deviation (*n* = 3) and that of in vivo assays were expressed as mean ± standard error (*n* = 6). The value of CHO cell control and CFCF control was taken as 100% for in vitro cytotoxicity and binding inhibition assays, respectively, and the value of test groups are represented as percentages relative to control group.

The difference of means between the control and test groups was analysed using One-Way ANOVA and Dunnett test (GraphPad Prism version 5) and * *p* < 0.05 was considered statistically significant. 

## Figures and Tables

**Table 1:** IC_50_ and two non-cytotoxic concentrations (NC1 and NC2) of seven extracts.

Plant Name	IC_50_ (µg/mL)	NC1 (µg/mL)	Viability (%)	NC2 (µg/mL)	Viability (%)
*Careya arborea* bark (CAE)	349.3 ± 19.3	85	90.1	42.5	95.1
*Punica granatum* peel (PGRPE)	446 ± 5.1	110	93.6	55	98.6
*Punica granatum* juice (PGRJ)	ND*	500	98.8	250	98.4
*Psidium guajava* leaf (PGAE)	486.4 ± 30.8	120	90.7	60	96.2
*Holarrhena antidysenterica* (HAE)	321.4 ± 22.1	80	89.9	40	91.2
*Aegle marmelos* fruit (AME)	646.3 ± 33.7	160	97.6	80	98.8
*Piper longum* fruit (PLE)	128.9 ± 14.2	35	91.3	17.5	96

ND*, Not determined.

## Data Availability

All the relevant data and the original contributions presented in the study are included in the manuscript. Further inquiries can be directed to the corresponding author.

## Supplementary Materials

The following supporting information can be downloaded at: https://www.mdpi.com/article/10.3390/toxins14100649/s1, Table S1: Extraction yield from six plants; Table S2: The mean W/L ratio of control and tested groups using adult mice ligated-ileal loop assay; Figure S1: IC_50_ of CFCF on CHO cell line using MTT assay.
