# Supplementary material for: In Vitro and In Vivo Inhibitory Activities of Selected Traditional Medicinal Plants against Toxin-Induced Cyto- and Entero- Toxicities in Cholera
Source: Toxins (Basel). 2022 Sep 20;14(10):649. doi: 10.3390/toxins14100649 (PMC9611792; doi:10.3390/toxins14100649)
Supplement: Supplementary file 1 [file toxins-14-00649-s001.zip › toxins-1876054-supplementary.pdf]

Supplementary Materials: In Vitro and In Vivo Inhibitory Activities of Selected Traditional Medicinal Plants Against Toxin-Induced Cyto- and En-Tero-Toxicities in Cholera

Rajitha Charla, Priyanka P. Patil, Arati A. Bhatkande, Nisha R. Khode, Venkanna Balaganur, Harsha V. Hegde, Darasaguppe R. Harish and Subarna Roy

Table S1. Extraction yield from six plants.

Name of the Plant (part)	Extraction Yield (%)
Careya arborea (bark)	7.67
Punica granatum (fruit peel)	5.83
Punica granatum (fruit juice)	6.87
Psidium guajava (leaf)	9.58
Holarrhena antidysenterica (bark)	8.24
Aegle marmelos (fruit)	4.86
Piper longum (fruit)	7.47

Table S2. The mean W/L ratio of control and tested groups using adult mice ligated-ileal loop assay.

Group Name	Mean W/L Ratio	SE
Saline control	0.078	0.011
CFCF control	0.249	0.023
CAE 100 µg	0.091	0.071
CAE 50 µg	0.184	0.040
PGRPE 100 µg	0.080	0.020
PGRPE 50 µg	0.190	0.053
PGAE 100 µg	0.150	0.078
PGAE 50 µg	0.209	0.042

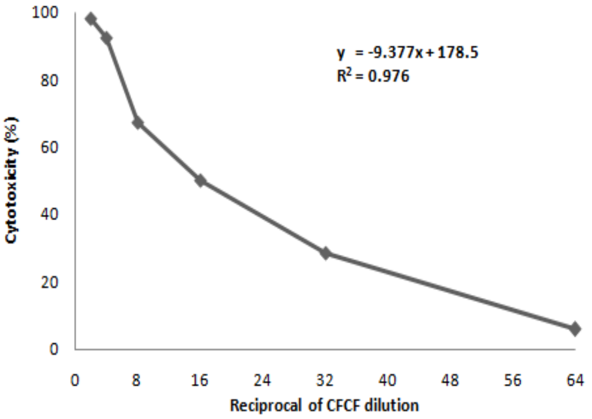

Figure S1. IC50 of CFCF on CHO cell line using MTT assay.
